# Evaluation of a newly developed piezo actuator-driven pulsed water jet system for liver resection in a surviving swine animal model

**DOI:** 10.1186/s12938-016-0126-9

**Published:** 2016-01-25

**Authors:** Chikashi Nakanishi, Toru Nakano, Atsuhiro Nakagawa, Chiaki Sato, Masato Yamada, Naoki Kawagishi, Teiji Tominaga, Noriaki Ohuchi

**Affiliations:** Division of Advanced Surgical Science and Technology, Graduate School of Medicine, Tohoku University, 1-1 Seiryou-machi, Aobaku, Sendai, 980-8574 Japan; Department of Neurosurgery, Graduate School of Medicine, Tohoku University, 1-1 Seiryou-machi, Aobaku, Sendai, 980-8574 Japan

**Keywords:** Pulsed water jet, Ultrasonic aspirator, Liver resection, Blood loss, Transection time

## Abstract

**Background:**

Preservation of the hepatic vessels while dividing the parenchyma is key to achieving safe liver resection in a timely manner. In this study, we assessed the feasibility of a newly developed, piezo actuator-driven pulsed water jet (ADPJ) for liver resection in a surviving swine model.

**Methods:**

Ten domestic pigs underwent liver resection. Parenchymal transection and vessel skeletonization were performed using the ADPJ (group A, *n* = 5) or an ultrasonic aspirator (group U, *n* = 5). The water jet was applied at a frequency of 400 Hz and a driving voltage of 80 V. Physiological saline was supplied at a flow rate of 7 ml/min. After 7 days, the animals were killed and their short-term complications were examined and compared between the two groups.

**Results:**

No significant complications, such as massive bleeding, occurred in either group during the surgical procedures. The transection time per transection area was significantly shorter in group A than in group U (1.5 ± 0.3 vs. 2.3 ± 0.5 min/cm^2^, respectively, *P* = 0.03). Blood loss per transection area was not significantly different between groups A and U (9.3 ± 4.2 vs. 11.7 ± 2.3 ml/cm^2^, *P* = 0.6). All pigs in group A survived for 7 days. No postoperative bleeding or bile leakage was observed in any animal at necropsy.

**Conclusion:**

The present results suggested that the ADPJ reduces transection time without increasing blood loss. ADPJ is a safe and feasible device for liver parenchymal transection.

## Background

Many interventions have been introduced to reduce intraoperative blood loss during liver surgery because excess blood loss increases the risk of postoperative complications [1]. Vascular occlusion techniques [2] and low central venous pressure anesthesia [3] are widely used to reduce bleeding during parenchymal resection. The introduction of hemostatic devices, such as the argon beam coagulator [4], ultrasonic coagulating shears [5], saline-linked radiofrequency technology [6, 7], and vascular staplers [8], has helped to limit blood loss during liver resection. The finger fracture technique was developed in the 1960s [9] to dissect the liver parenchyma while preserving the hepatic vessels. This was followed by the introduction of the clamp crushing method [10], ultrasonic aspirators (UA) [11], and water jet dissectors with continuous water flow [12, 13]. However, the optimal method for liver parenchymal resection while preserving the hepatic vessels, to minimize blood loss and procedure time, remains to be established. Currently, the type of parenchymal dissection method used during surgery is largely at the surgeon’s preference.

Water jet dissectors with continuous water flow allow the surgeon to dissect an organ while preserving vessels exceeding 100–200 μm in diameter [14, 15]. Nevertheless, there are some limitations to these tools, particularly the formation of air bubbles, which obscure the operative field, and the splashing of bloody fluids, which could increase the risk of cross-infection to surgeons and nurses [15]. Pulsed water jet dissection is an emerging technology that enables tissue dissection with markedly reduced water consumption [16]. Therefore, this technology could reduce bubble formation and splashing, for example. In recent neurosurgical studies using laser-induced pulsed water jet devices, the investigators significantly reduced the intraoperative blood loss and procedure times while increasing the tumor volume removed from patients with complex pituitary and skull-base lesions [17].

The piezo actuator-driven pulsed water jet system (ADPJ) is a new technology that emits a minimal amount of stably pulsed water, and allows fine control of the dissection in soft-tissue phantoms [16]. In an ex vivo study using swine livers, this system allowed the operator to dissect the liver parenchyma, while preserving the hepatic veins and Glisson’s sheaths. The peak pressure of the pulsed water jet was controlled by changing the input voltage [18].

The aim of the present study was to assess the feasibility, efficacy, and safety of a new method of liver resection using the ADPJ, and to compare it with a widely used device (i.e. UA) in a surviving swine model.

## Methods

### Overview of the ADPJ (Fig. 1)

The design and features of the ADPJ have been described in more detail elsewhere [18]. Briefly, the ADPJ consists of a handpiece (254 mm long; Fig. 2), a supply pump, and a controller. The piezo actuator (3.5 mm × 3.5 mm × 18 mm; model PSt 150/3.5 × 3.5/20; Piezomechanik GmbH, Munich, Germany), which has a displacement of 20 μm without a payload at an input voltage of 150 V, is glued to an aluminum disk (5.7 mm in diameter and 0.7 mm thick), which acts as a piston. The piston is glued to a stainless steel diaphragm (0.02 mm thick) and fixed onto the metal wall at its margin, but is separated at its center by 0.1 mm from the metal wall, so that it can feed water into the chamber (height of 0.1 mm). A straight stainless steel connecting pipe with an internal diameter of 1.1 mm is connected to the actuator. A nozzle (0.15 mm in diameter) is attached to the connecting pipe. A supply pump continuously feeds physiological saline into the chamber at a controllable flow rate through a capillary inlet (0.3 mm in diameter). To drive the piston, a voltage is applied to the piezo actuator at a specific frequency. The pulsed water jet is therefore ejected at a frequency-based pulse rate.

**Fig. 1:**
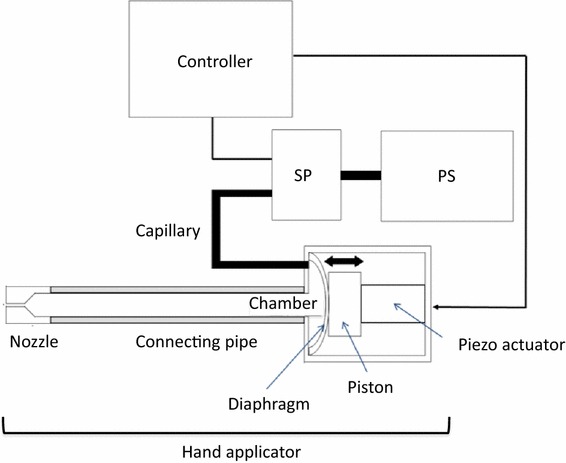
Schematic diagram of the piezo actuator-driven pulsed water jet system. The system consists of a handpiece, a supply pump, and a controller. The supply pump continuously feeds physiological saline into the chamber through a capillary inlet. To drive the piston, a voltage is applied from the controller to the piezo actuator at a specific frequency. The pulsed water jet is ejected at a frequency-based pulse rate. *SP* supply pump, *PS* physiological saline

**Fig. 2:**
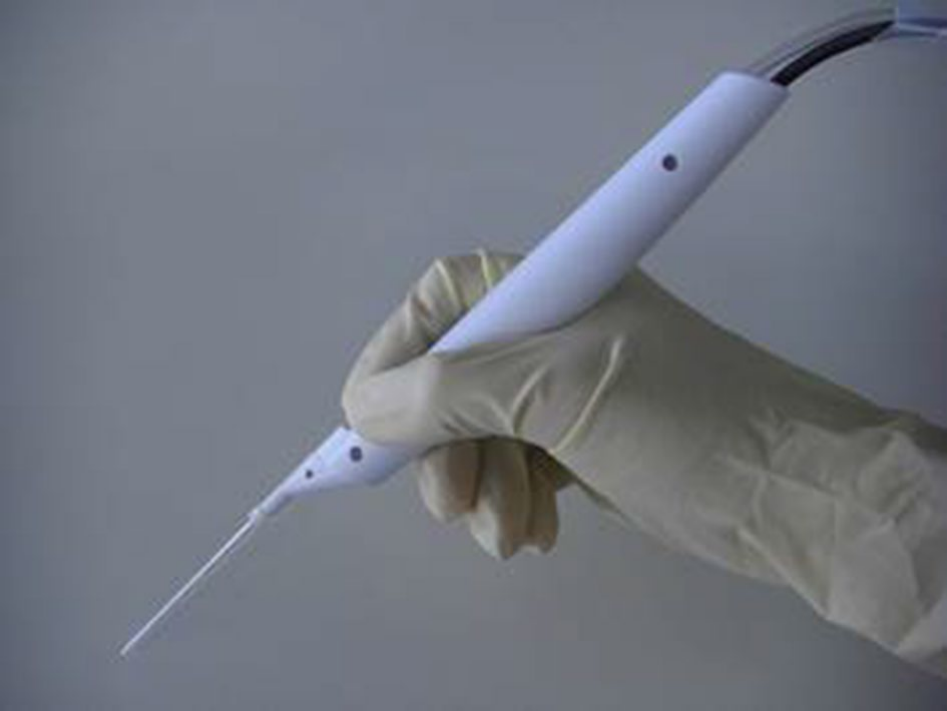
The handpiece of the piezo actuator-driven pulsed water jet. The total length of the handpiece is 254 mm. The nozzle is 0.15 mm in diameter

### Animals

Ten domestic pigs (*Sus scrofa domesticus* LWD, two males and eight females) with a mean weight of 36.0 kg (range 27.3–42.0 kg) were used in this study. All animal procedures and protocols were approved by the Institutional Review Board at the Center for Laboratory Animal Research, Tohoku University (the approval code: 21-idou-269).

### ADPJ settings

The pulsed water jet was applied at a frequency of 400 Hz and a driving voltage of 80 V. Physiological saline was supplied at a flow rate of 7 ml/min. The individual parameters were determined in preliminary experiments in which various driving voltages and flow rates were tested during the dissection of swine livers (data not shown).

### Anesthesia

The animals were anesthetized with 0.04–0.06 mg/kg of medetomidine chloride, 0.2–0.4 mg/kg of midazolam, and 0.2 mg of buprenorphine hydrochloride. Isoflurane gas was used to maintain anesthesia during the procedure (model PH-3F; Acoma Co., Tokyo, Japan) with mechanical ventilation (ARF-900; Acoma Co.). Heart rate, oxygen saturation, two-lead electrocardiography, and arterial blood pressure were continuously monitored throughout the procedure. Central venous pressure was measured at the start and the end of the liver resection, and was about 5 mmHg. Postoperative pain was controlled with rectal buprenorphine and acetaminophen.

### Surgical procedures

**Fig. 3:**
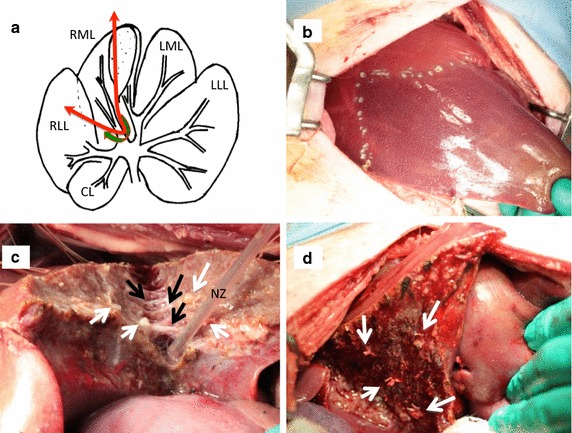
Surgical procedures. **a** The Glissonean pedicle of the right inferior side of the right median lobe (segment V and a part of segment VIII) was encircled and ligated at the hepatic hilum without liver dissection (*white arrow*). After the border between the ischemic area and the residual area was confirmed, the liver parenchyma was dissected (*black arrow*). *LLL* left lateral lobe, *LML* left median lobe, *RML* right median lobe, *RLL* right lateral lobe, *CL* caudate lobe. **b** The border between the ischemic area and the residual area was apparent after ligation of the Glissonean pedicle. **c** When the piezo actuator-driven pulsed water jet system was used to transect the liver parenchyma, the vessels were clearly skeletonized with little damage (*black arrows*). Some vessels were ligated (*white arrows*). *NZ* nozzle of the handpiece. **d** Hemostasis was achieved when the transection was complete. Some vessels were ligated (*white arrows*)

The same surgeon performed all of the liver resections. The liver was accessed by a midline laparotomy. After cholecystectomy, the right inferior side of the right median lobe (segment V and a part of segment VIII) was resected (Fig. 3a). The Glissonean pedicle of these segments was encircled and ligated at the hepatic hilum, without liver dissection. The liver parenchyma was dissected after the surgeon had confirmed the border between the ischemic area and residual area (Fig. 3b). All resections were performed with temporary vascular occlusion (Pringle maneuver). Vascular occlusion and liver resection were stopped if the arterial systolic blood pressure dropped below 80 mmHg or oxygen saturation dropped below 90 %. Vascular occlusion and liver resection were resumed once the arterial systolic blood pressure and oxygen saturation had recovered.

### Experimental design

The 10 animals were randomly allocated to two groups. The capsule of the liver was dissected with an electrosurgical knife (Valleylab SurgiStat™ II, Coviden Japan Co., Tokyo, Japan) in each animal. Transection of the parenchyma and skeletonization of the vessels were performed with the ADPJ (group A, *n* = 5) (Fig. 3c) or with an UA (SonoSurg^®^; Olympus Corporation, Tokyo, Japan; group U, *n* = 5). Small vessels (usually <1 mm in diameter) were cut and cauterized with an electrosurgical knife, and large vessels were ligated before transection.

### Outcome measures

Blood loss was calculated by adding the volume of the soaked gauze swabs to the amount of blood collected in the containers of the suction apparatus and the ADPJ or UA. The volume of saline instilled using the ADPJ or UA was then subtracted from this volume. The duration of liver transection was measured from the start to the end of parenchymal transection, excluding the waiting time for the Pringle maneuver. Thus, the transection time corresponds to the Pringle maneuver time. Immediately after parenchymal transection was completed, the transected surface of the removed specimen was traced onto paper and digitally photographed together with a ruler. The transection area was then calculated using cellSens Standard 1.5 software (Olympus Co., Tokyo, Japan). The transection time per transection area and blood loss per transection area were also calculated.

On postoperative day 7, the abdomen was inspected for short-term intra-abdominal complications and the animals were then killed with an overdose of potassium chloride.

### Measurement of serum electrolytes

Blood samples were collected from each animal before surgery and after completing liver resection. Sera were prepared by centrifuging the blood samples at 1750×*g* for 15 min at 4 °C, followed by filtration. After filtration, the serum electrolyte concentrations (sodium and chloride) were measured (testing was conducted by Mitsubishi Chemical Medience Co., Tokyo, Japan).

### Histopathological evaluation

The resected liver was fixed in formaldehyde and embedded in paraffin using routine methods. The histopathological characteristics of resected tissue samples were assessed using hematoxylin and eosin-stained sections.

### Statistical analysis

Data are presented as means ± standard deviations. Mean values were compared between the two groups using Student’s *t* test for parametric variables or Wilcoxon rank sum test for non-parametric variables. Shapiro–Wilk test was used to assess whether the variables had a parametric distribution. Differences between the two groups were considered significant at a threshold of *P* < 0.05. The sex of the animals was not considered a factor in the statistical analysis of the data. Statistical analyses were performed with JMP^®^ 10 (SAS Institute Inc., Cary, NC, USA).

## Results

### Surgical outcomes

During parenchymal resection, the vessels were clearly skeletonized using the ADPJ, with little damage (Fig. 3c, d). The central venous pressure was not significantly different between groups A and U (5.3 ± 1.7 vs. 6.1 ± 2.7 mmHg, respectively, *P* = 0.6). All the animals tolerated the procedures and no major complications, such as massive bleeding, occurred during the surgical procedures in either group. However, one pig in group U died several hours after surgery. This pig tolerated the surgical procedures. The blood loss volume in this pig was 362.4 ml, which was not excessive compared with the mean blood loss in the other 9 pigs (359.8 ± 159.4 ml). However, the pig’s oxygen saturation and arterial blood pressure decreased at the end of surgery. After it recovered from anesthesia, its blood pressure and oxygen saturation recovered. It was transferred to the recovery room after successful extubation. The pig died in the recovery room several hours after surgery. There was no sign of postoperative bleeding or bile leak at necropsy. The cause of death remains unclear. The other four pigs in group U and all the pigs in group A survived for 7 days without postoperative bleeding or bile leakage (Table 1).

**Table 1:** Intraoperative variables

	Group A^a^ (n = 5)	Group U^b^ (n = 5)	*P* value
Transection time (min)	57.2 ± 7.9	69.4 ± 10.4	0.07
Transection area (cm^2^)	38.4 ± 6.9	31.7 ± 8.7	0.2
Weight of resected tissue (g)	83.9 ± 36.1	73.3 ± 36.2	0.7
Blood loss (ml)	343 ± 121	384 ± 188	0.7
Transection time per transection area (min/cm^2^)	1.5 ± 0.3	2.3 ± 0.5	0.03
Blood loss per transection area (ml/cm^2^)	9.3 ± 4.2	11.7 ± 2.3	0.6
Instilled saline volume (ml)	119 ± 35	98 ± 35	0.4
Intraoperative complications	0	0	
Postoperative complications	0	1 (unexplained death)	

Values are presented as the mean ± standard deviation or *n*

^a^Piezo actuator-driven pulsed water jet

^b^Ultrasonic aspirator

### Intraoperative transection-related outcomes

The transection area (38.4 ± 6.9 vs. 31.7 ± 8.7 cm^2^, respectively, *P* = 0.2) and resected liver weight (83.9 ± 36.1 vs. 73.3 ± 36.2 g, respectively, *P* = 0.7; Table 1) were not significantly different between groups A and U. The transection time was shorter in group A (57.2 ± 7.9 min) than in group U (69.4 ± 10.4 min), although the difference was not statistically significant (*P* = 0.07). Furthermore, the transection time relative to the transection area was significantly different between groups A and U (1.5 ± 0.3 vs. 2.3 ± 0.5 min/cm^2^, respectively, *P* = 0.03). Blood loss (343 ± 121 vs. 384 ± 188, respectively, *P* = 0.7) and blood loss per transection area (9.3 ± 4.2 vs 11.7 ± 2.3 ml/cm^2^, respectively, *P* = 0.6) were not significantly different between groups A and U (Table 1). The mean volume of saline instilled was not significantly different between groups A and U (119 ± 35 vs. 98 ± 35 ml, respectively; *P* = 0.2) (Table 1).

### Serum sodium and chloride concentrations (Table 2)

Serum sodium and chloride concentrations decreased slightly after liver resection in all of the animals, except for two in group A, respectively. Although the serum sodium concentrations after liver transection were higher in group A than in group U, the changes in serum sodium concentration (−1.4 ± 2.6 vs. −2.8 ± 1.5 mEq/l, respectively, *P* = 0.3) and serum chloride concentrations (−1.0 ± 2.2 vs. −3.4 ± 0.5 mEq/l, respectively, *P* = 0.09) from before to after surgery were not significantly different between groups A and U.

**Table 2:** Serum sodium and chloride concentrations

	Serum sodium, mEq/l (RV: 139–153 mEq/l)			Serum chloride, mEq/l (RV: 97–106 mEq/l)		
	Before resection	After resection	Change	Before resection	After resection	Change
Group A^a^ (n = 5)	144.8 ± 5.1	143.4 ± 5.2	−1.4 ± 2.6	103.0 ± 2.2	102.0 ± 3.7	−1.0 ± 2.2
Group U^b^ (n = 5)	139.8 ± 0.8	137.0 ± 2.0	−2.8 ± 1.5	101.8 ± 1.3	98.4 ± 1.8	−3.4 ± 0.5
*P* value	0.09	0.03	0.3	0.3	0.09	0.09

*RV* reference value

^a^Piezo actuator-driven pulsed water jet

^b^Ultrasonic aspirator

### Histopathological findings (Fig. 4)

**Fig. 4:**
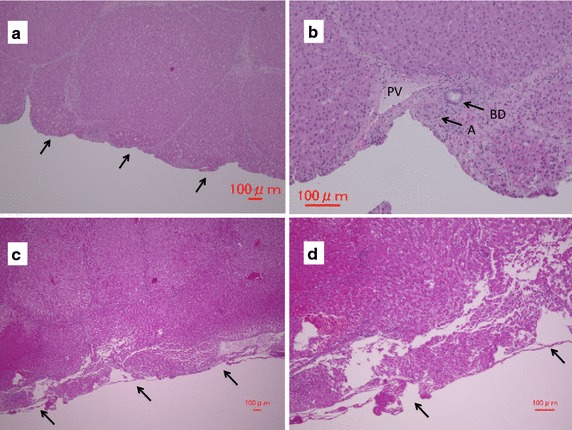
Histopathological features of the transected surface. Haematoxylin-Eosin staining. All* scale bars* represent 100 µm. **a** The liver parenchyma was sharply dissected (*arrows*) without damage by the piezo actuator-driven pulsed water jet (ADPJ). **b** The cut surface of the liver dissected with the ADPJ. Glisson’s sheaths, including those covering the portal vein, artery, and bile duct, were preserved. *PV* portal vein, *A* artery, *BD* bile duct. **c** The cut surface of the liver dissected with the ultrasonic aspirator (*arrows*) was blunter than that dissected with the ADPJ. **d** The surface cut with the ultrasonic aspirator shows parenchymal damage, including the loss of hepatocytes (*arrows*)

The histopathological analysis revealed that the liver parenchyma was dissected with the ADPJ while preserving the Glisson’s sheaths, including those covering the portal vein, artery, and bile duct (Fig. 4a, b). The cut surface of the liver dissected with the UA appeared to be blunter than the surface cut with the ADPJ, and a loss of hepatocytes was observed in group U (Fig. 4c, d).

## Discussion

In this study, liver resection was safely performed with ADPJ in a swine model. One animal died after liver resection with UA, and the cause of death remains unknown. However, all the animals that underwent parenchymal resection with the ADPJ survived for 7 days and suffered no surgical complications during or after liver resection. Although blood loss was not significantly different between the two groups, the transection time relative to the transection area was shorter using the ADPJ than using the UA. These findings suggest that the ADPJ can reduce transection time without increasing blood loss, compared with the UA.

Until now, only a few controlled studies have compared the intraoperative blood loss or transection time between various devices used for liver parenchymal dissection, such as the UA and water jet. Takayama et al. [19] concluded from the results of a randomized clinical trial that the use of UA was not superior to the clamp crushing method in reducing intraoperative blood loss or transection time. They also reported that the quality of resection was inferior using the UA compared with the clamp crushing method. Some surgeons have used water jet devices with a continuous water flow for open and laparoscopic liver resection, and have reported that water jet devices reduced blood loss, transection time, and complication rates compared with other conventional methods, such as the UA [12, 13]. Stapler hepatectomy is known to be one of the fastest liver resection devices [8]. However, stapler resection cannot expose the hepatic vein or Glissonean pedicle during liver resection such as right hepatectomy or sectionectomy. Although further research is required to compare the ADPJ with other devices, such as water jet devices with a continuous water flow, we suggest that the ADPJ is a useful device for liver resection while retaining the advantages of water jets, particularly reduced blood loss and shorter transection time.

Because the ADPJ uses saline, we measured the serum sodium and chloride concentrations before and after resection. Notably, we found no disturbances in the serum sodium or chloride concentrations. Furthermore, the changes in serum sodium and chloride concentrations between before and after liver resection were similar between the ADPJ and the UA. These results suggested that liver resection with the ADPJ does not cause specific electrolyte changes.

The water jet might also be useful for endoscopic surgery. Sato et al. reported that an endoscopic pulsed water jet system is a feasible alternative to endoscopic submucosal dissection in a swine model [20]. Likewise, Shi et al. [21] have safely performed natural orifice transluminal endoscopic hepatic resection with a water jet device in a non-surviving swine model.

The ADPJ could overcome some of the problems associated with water jet devices that arise from the continuous water flow. The peak pressure of the pulsed water jet is controllable in the ADPJ by changing the input voltage [18]. Therefore, this device can be used to dissect various tissues with different physical properties. Thus, the ADPJ seems to be suitable for use in human liver resection because of differences in the physical properties of the human liver parenchyma between healthy and diseased states, such as hepatic steatosis or fibrosis.

## Conclusion

We successfully performed liver resection with a new system, the ADPJ, in a surviving swine model. Our results suggest that like UA, the ADPJ is a safe and feasible tool for liver parenchyma resection. The ADPJ has great potential in a variety of settings, and future studies should investigate the use of this system in natural orifice transluminal endoscopic hepatic resection and robotic surgery.

## Abbreviations

ADPJ: piezo actuator-driven pulsed water jet
UA: ultrasonic aspirator
